# Chromium(III) Beyond Insulin: A Mechanistic Roadmap Across the Energy–Redox–Inflammation Triad in Obesity and Cardiometabolic Disease

**DOI:** 10.1007/s12011-026-05161-y

**Published:** 2026-05-25

**Authors:** Mehmet Tuzcu, Ramazan Ozmen, Kazim Sahin

**Affiliations:** 1 Department of Biology, Faculty of Science, Firat University, Elazig, 23119 Türkiye; 2 Department of Animal Nutrition and Nutritional Diseases, Faculty of Veterinary Medicine, Firat University, Elazig, 23119 Türkiye

**Keywords:** Chromium(III), AMP-activated protein kinase, Nrf2/HO-1 pathway, NF-κB, Obesity

## Abstract

This review advances a framework that moves beyond an “ultrainsulinocentric” view of chromium(III) (CrIII) in obesity toward an integrated model centered on mitochondrial energy sensing, redox homeostasis, and inflammatory regulation. Obesity is now recognized as a systemic disorder marked by chronic low-grade inflammation and metabolic dysregulation driven by impaired AMPK activity, disrupted Nrf2/HO-1 signaling, and persistent NF-κB–mediated metaflammation. Traditional models that depict CrIII as a direct enhancer of insulin signaling are insufficient to account for its disputed essentiality, variable clinical efficacy, and emerging toxicological concerns. We synthesize mechanistic, toxicological, and clinical evidence, emphasizing ATP synthase-AMPK signaling, Nrf2/HO-1 activation, NF-κB inhibition, and classical insulin pathways in obesity, type 2 diabetes, and nonalcoholic fatty liver disease. Experimental data suggest that CrIII may accumulate within mitochondria, partially inhibit ATP synthase, and activate AMPK, thereby enhancing fatty acid oxidation and limiting lipogenesis. Concurrently, CrIII appears to strengthen antioxidant defenses and suppress inflammatory signaling, indirectly improving insulin sensitivity. Some clinical trials report modest improvements in glycemic and inflammatory markers, with substantial heterogeneity related to phenotype, formulation, dose, and duration. We propose that CrIII acts as a context-dependent modulator of the energy-redox-inflammation axis. Current clinical evidence remains insufficient to validate this mechanistic framework; future biomarker-driven, phenotype-stratified trials are required to determine whether modulation of these pathways mediates clinical benefit.

**Graphical Abstract:**

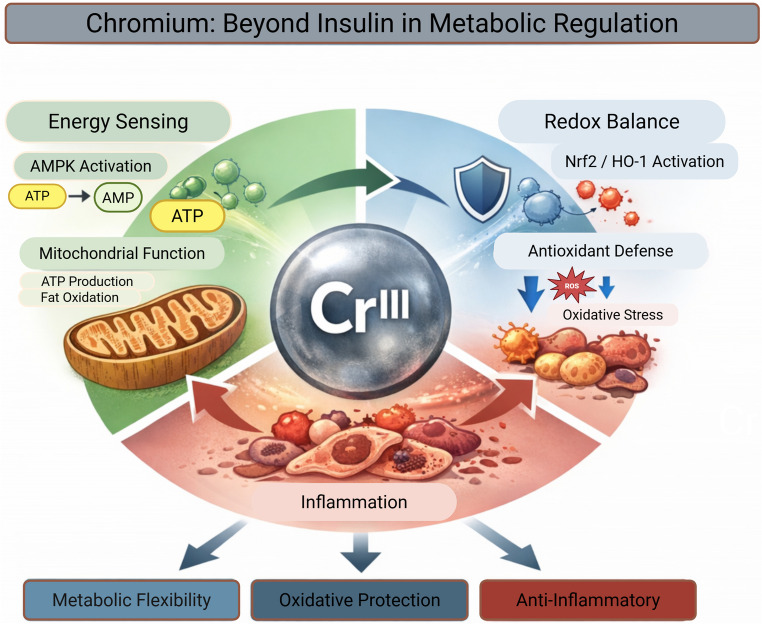

## Introduction

Obesity is increasingly recognized as a complex systemic disorder characterized by mitochondrial dysfunction, impaired cellular energy sensing, oxidative stress, and chronic low-grade inflammation, rather than a passive consequence of excess energy intake [1–3]. These interconnected processes contribute to metabolic inflexibility and impaired insulin action in key tissues, including the liver, muscle, and adipose tissue [2]. Central regulatory nodes, including AMP-activated protein kinase (AMPK), nuclear factor erythroid 2–related factor 2 (Nrf2), and nuclear factor-κB (NF-κB), coordinate the integration of energy status, redox balance, and inflammatory signaling within this network [4–6]. In this context, chromium(III) (CrIII) has historically been investigated as a modulator of insulin signaling. Early experimental studies suggest that chromium supplementation may enhance insulin action, including modulation of IRS-mediated PI3K/Akt signaling and increased GLUT4 expression, thereby promoting glucose uptake in insulin-sensitive tissues, although these effects appear to be model-dependent [7–10]. Chromium biology has historically been interpreted within the context of physiological stress and impaired metabolic control rather than universal metabolic enhancement. Pioneering studies by Mertz and Anderson [11] demonstrated that the beneficial effects of chromium(III) supplementation were most evident in experimental settings characterized by metabolic perturbation, including impaired glucose tolerance, oral glucose tolerance testing (OGTT), inadequate chromium intake, or nutritional stress. In controlled low-chromium diets, chromium supplementation improved glucose, insulin, and glucagon responses following OGTT in subjects with impaired glucose handling, whereas metabolically healthy individuals showed minimal or no response. Similarly, early diabetic studies reported improvements in glucose tolerance predominantly in metabolically compromised individuals rather than normoglycemic controls [12]. Subsequent reviews by Mertz further emphasized that chromium-responsive phenotypes are typically associated with disturbed metabolic states, including insulin resistance, malnutrition, or stress-related metabolic dysfunction [13]. This historical framework is highly relevant to contemporary obesity and cardiometabolic research, as it suggests that chromium should not be viewed as a universal insulin sensitizer or essential nutrient for healthy individuals, but rather as a context-dependent modulator whose biological effects emerge primarily under conditions of energetic, oxidative, inflammatory, or metabolic stress. These findings led to the longstanding view of chromium as a metabolically important trace element. However, this framework remains mechanistically incomplete and clinically controversial.

A major limitation is the absence of reliable biomarkers of chromium nutritional status and the lack of a clearly defined deficiency phenotype across both humans and animal species, which challenges its classification as an essential nutrient [9, 14]. Although serum chromium concentration is commonly used as a proxy for nutritional chromium intake, it does not reliably reflect tissue stores or functional chromium activity, and its association with metabolic impairment remains uncertain [15]. In this context, “chromium status” refers to the integrated assessment of chromium intake, tissue availability, and functional biological activity; however, no validated biomarker currently captures these dimensions. This position is further supported by the Nordic Nutrition Recommendations 2023, which concluded that the available evidence is insufficient to establish dietary reference values for chromium due to the lack of defined criteria of essentiality [15]. Clinical evidence presents a complex and often inconsistent picture, as although several meta-analyses report significant improvements including glycemic control, and lipid profiles, following chromium supplementation, these effects are generally modest and of uncertain clinical relevance, while other analyses yield inconsistent or null findings, reflecting substantial heterogeneity in study design, populations, baseline metabolic status, dosage, duration, and chromium formulations [16–21]. However, this heterogeneity does not provide support for any specific molecular mechanism. While inter-individual variability has been proposed as a contributing factor, this remains speculative; alternative explanations include minimal true biological effect as well as methodological limitations such as small sample sizes, heterogeneity, and potential bias in clinical trials. Collectively, these uncertainties challenge not only the insulin-centric model but also the adequacy of any single-mechanism framework to explain chromium’s metabolic effects [9, 15, 16, 21, 22]. Accordingly, the role of CrIII in obesity pathophysiology remains uncertain and cannot be reliably attributed to a single mechanism, including insulin signaling. Although experimental studies suggest potential interactions with cellular energy sensing and stress-response pathways, the available evidence is inconsistent and does not support a definitive mechanistic role [14, 16, 21]. A recent study has identified the mitochondrial ATP synthase β-subunit as a potential molecular target of CrIII, suggesting a possible link between chromium and cellular energy metabolism outside insulin signaling pathways [23]. In this model, modulation of ATP synthase could alter the AMP/ATP ratio and activate AMPK, a key regulator of metabolic homeostasis. However, this mechanism should be interpreted with caution. The evidence is primarily derived from in vitro and experimental systems, and the intracellular chromium concentrations required for ATP synthase inhibition may not be physiologically achievable in vivo. Therefore, while AMPK activation remains a plausible contributor, it may occur through indirect or alternative pathways rather than direct ATP synthase inhibition [23, 24]. In parallel, experimental evidence indicates that chromium may influence redox and inflammatory pathways. Studies in high-fat diet models demonstrate that chromium supplementation can enhance Nrf2/HO-1 signaling and attenuate NF-κB activation and lipid peroxidation, suggesting a potential role in modulating oxidative stress and inflammatory responses [25–27]. Given that obesity is characterized by increased reactive oxygen species production and persistent NF-κB activation, these pathways represent key targets linking metabolic dysfunction with inflammation.

The novelty of this review is to relocate CrIII from the few known insulin-sensitizing and trace-element carriers to a multi-level controller of cellular stress and energy signaling. By incorporating new findings on ATP synthase-AMPK cross-talk alongside established data on Nrf2/HO-1 activation and NF-κB inhibition, this study presents a holistic systems-level framework of chromium’s actions in the obese state. This “beyond insulin” paradigm is not only consistent with, but also provides testable hypotheses for the mechanistic underpinnings of intermediate phenotypes in future biomarker-driven trials in the field of chromium within modern metabolic and mitochondrial biology frameworks, rather than archaic micronutrient paradigms. Table 1 summarizes the rationale for broadening CrIII biology beyond an exclusively insulin-centric model to incorporate energy sensing, redox regulation, and inflammation control. The absence of validated biomarkers and established dietary reference values (DRVs), coupled with modest and inconsistent clinical efficacy, challenges the classification of CrIII as an essential nutrient and instead supports its role as a context-dependent modulator of metabolic function.

**Table 1:** Conceptual rationale for adopting a “beyond insulin” framework in chromium(III) biology

Justification	Key Findings	References
Uncertain essentiality	No reliable biomarker; insufficient evidence for DRV recommendation	[9, 15]
Heterogeneous clinical effects	Small to moderate glycemic and lipid responses; highly variable outcomes	[14, 16, 89]
Oxidative stress and inflammation	NF-κB inhibition, reduced ROS, increased TAC	[73, 88]
Energy and redox nodes	Proposed modulation of AMPK, Nrf2, and mitochondrial targets	[10, 23, 24]

*DRV* dietary reference value, *AMPK* AMP-activated protein kinase, *Nrf2* nuclear factor erythroid 2–related factor 2, *NF-κB* nuclear factor kappa-light-chain-enhancer of activated B cells, *ROS* reactive oxygen species, *TAC* total antioxidant capacity

### A Mechanistic Roadmap: The Energy-Redox-Inflammation Triad

In the context of obesity, the proposed energy-redox-inflammation triad offers a common mechanistic schema, previously described solely in terms of insulin-oriented interpretations, and brings together mitochondrial energetic disarray, antioxidant defense system activity, and nuclear inflammatory transcriptional dominance [28]. Rather than targeting a single robust pathway, CrIII appears to modulate obesity-relevant physiology by coordinately regulating these interlinked modules [29]. Although the factors have typically been considered separately in previous studies, an accumulating body of evidence indicates that functional convergence among these factors may be more pertinent for achieving a better understanding of the context-dependent metabolic actions of chromium in obesity and cardiometabolic disease [29]. This context dependency is consistent with the classical observations of Mertz and Anderson [11], who reported that chromium responsiveness was most apparent under conditions of physiological or metabolic stress, including impaired glucose tolerance, low chromium intake, or nutritional imbalance. In this regard, the proposed ATP synthase–AMPK/Nrf2/NF-κB framework may be particularly relevant in stressed metabolic states characterized by mitochondrial dysfunction, oxidative stress, inflammatory activation, or impaired insulin responsiveness, rather than in metabolically healthy systems.

Within this framework, three interrelated regulatory axes are particularly important: ATP synthase–AMPK signaling as a determinant of cellular energy sensing, Nrf2/HO-1 signaling as a core antioxidant and cytoprotective pathway, and NF-κB as a central mediator of metaflammation. These systems connect mitochondrial dysfunction, oxidative stress, and chronic low-grade inflammation, hallmarks of obesity and insulin resistance. Positioning chromium biology within this triad helps move the discussion beyond a narrow insulin-centered model and offers a broader conceptual framework for contextualizing the experimental findings, while acknowledging that the clinical relevance of these pathways has not yet been established.

### ATP Synthase–AMPK Cross-talk: Energy Sensing and Metabolic Switching

**Fig. 1:**
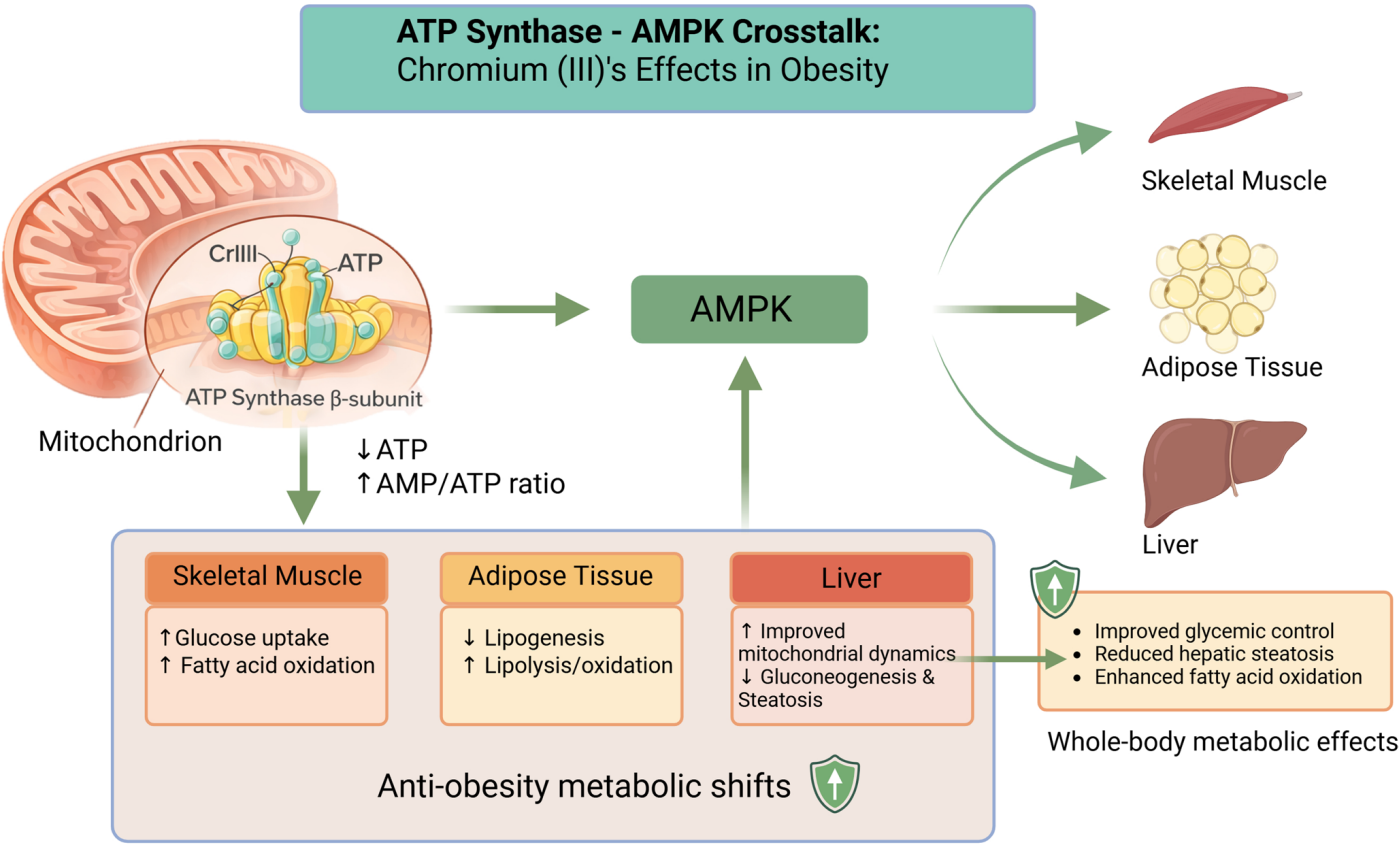
ATP synthase–AMPK cross-talk as an upstream mechanism of chromium(III) action in obesity. Chromium(III) (CrIII) binds to the β-subunit of mitochondrial ATP synthase, inducing a mild reduction in ATP production and increasing the AMP/ATP ratio. The resulting energetic shift activates AMP-activated protein kinase (AMPK), which reprograms metabolism across skeletal muscle, adipose tissue, and liver. In skeletal muscle, AMPK enhances glucose uptake and fatty acid oxidation. In adipose tissue, it suppresses lipogenesis while increasing lipolysis and fatty acid oxidation. In the liver, AMPK improves mitochondrial dynamics and reduces gluconeogenesis and steatosis

AMPK is the principal cellular energy sensor and is activated when the AMP/ATP ratio rises under conditions of energetic stress. In obesity and related cardiometabolic disorders, AMPK activity is commonly suppressed in metabolically active tissues such as the liver, skeletal muscle, adipose tissue, and heart, thereby favoring de novo lipogenesis, ectopic lipid deposition, mitochondrial dysfunction, and inflammatory signaling [30–32]. A major unresolved question in chromium biology has been how CrIII might engage this pathway. A recent mechanistic study identified the mitochondrial ATP synthase β-subunit (ATP5B) as a potential molecular target of CrIII, suggesting a link between chromium exposure and AMPK activation via modulation of cellular energy status; however, the physiological relevance and generalizability of this mechanism remain uncertain [23] (Fig. 1). Conceptually, this resembles mild mitochondrial-derived energetic stress, by which metformin activates AMPK rather than causing overt mitochondrial toxicity [33]. This mechanism, therefore, suggests an insulin-independent entry point through which CrIII may influence metabolic regulation in obesity, where canonical insulin signaling is often impaired [23, 24, 29].

Once activated, AMPK initiates a coordinated metabolic response that opposes several hallmarks of obesity. In skeletal muscle, it enhances glucose uptake through GLUT4 translocation, promotes fatty acid oxidation through ACC phosphorylation, and improves mitochondrial biogenesis and oxidative capacity [32, 34–36]. In the liver, AMPK suppresses gluconeogenesis and restrains lipogenesis by modulating regulators such as CRTC2, SREBP-1c, ChREBP, and HNF4α, thereby reducing hepatic glucose output and steatosis while improving insulin sensitivity [30, 32, 33, 37]. In adipose tissue, it promotes mitochondrial biogenesis, fatty acid oxidation, and browning-associated thermogenic programs, whereas AMPK deficiency contributes to obesity and metabolic dysfunction [30, 31, 38].

Experimental evidence indicates that ATP synthase–targeted CrIII increases AMPK phosphorylation, improves glucose metabolism, and attenuates hyperglycaemia-induced mitochondrial fragmentation in hepatocytes and diabetic db/db mice; however, these findings are primarily derived from experimental models and their systemic physiological relevance remains to be established [23]. Some chromium compounds, such as chromium picolinate, can activate AMPK, and their metabolic effects, including improvements in GLUT4 translocation and glucose uptake, appear to be AMPK-dependent, as demonstrated by siRNA-mediated AMPK knockdown experiments [39]. However, this effect is not consistent across all chromium complexes. In oligomannuronate–CrIII systems, the observed activity is largely attributable to the ligand rather than chromium itself: CrCl₃ alone shows no significant effect on PGC1α transcription, ATGL expression, or AMPK phosphorylation, while the ligand alone remains biologically active, and in some studies, appropriate inorganic chromium controls are absent [40, 41]. Mechanistic interpretations attributing these effects specifically to chromium should therefore be made with caution, particularly in the absence of appropriate controls.

### Nrf2/HO-1 Module: Antioxidant and Cytoprotective Responses

Obesity is increasingly recognized as a state of redox imbalance characterized by excessive reactive oxygen species (ROS) generation, lipid peroxidation, and impaired antioxidant defense. These abnormalities contribute to mitochondrial dysfunction, insulin resistance, and inflammatory signaling in tissues such as the liver, skeletal muscle, and adipose tissue [25, 42, 43]. In this setting, the Nrf2/HO-1 axis represents one of the major adaptive cytoprotective systems. Under basal conditions, Nrf2 is sequestered in the cytosol by Keap1 and targeted for proteasomal degradation. Oxidative or electrophilic stress disrupts this interaction, allowing Nrf2 to translocate to the nucleus and activate antioxidant response element (ARE)-driven genes encoding HO-1, NQO1, superoxide dismutase, catalase, glutathione-related enzymes, and other phase II detoxifying proteins [43–46]. HO-1 is particularly important because degradation of pro-oxidant heme generates biliverdin/bilirubin and carbon monoxide, conferring antioxidant, anti-apoptotic, and anti-inflammatory effects while also influencing mitochondrial and inflammatory signaling [43, 45].

Experimental evidence suggests that chromium may modulate this pathway in obesity-related models. In high-fat diet-fed rats, chromium histidinate markedly increased hepatic Nrf2 and HO-1 protein levels while reducing oxidative stress markers and lipid peroxidation products, including 4-hydroxynonenal (4-HNE), together with improvements in glucose and insulin indices [25]. Similarly, chromium picolinate and nanoparticulate chromium have been associated with improved hepatic GSH/GSSG balance, reduced COX-2 and LOX-1 expression, attenuation of steatosis and inflammation, and restoration of the Nrf2/NF-κB balance [27].

### Nf-κB Suppression: Attenuation of Inflammatory Transcription

Chronic low-grade inflammation is a defining feature of obesity and exposure to a high-fat diet, and NF-κB is a central transcriptional regulator of this process. Its activation promotes cytokine production, insulin resistance, and tissue injury, thereby linking metabolic stress to regulation of organ pathology [47, 48]. Because oxidative stress and mitochondrial dysfunction are major upstream drivers of NF-κB, inflammatory control is mechanistically intertwined with both energy sensing and redox regulation. A number of chromium preparations have been reported to attenuate NF-κB signaling in obesity-related models. Chromium histidinate suppresses NF-κB p65 nuclear activity and downstream inflammatory signaling in metabolically relevant tissues such as the liver in high-fat diet-induced obesity [25, 49]. Other chromium formulations, including chromium–D-phenylalanine complexes, have been associated with reduced TNF-α and improvements in inflammatory and histological parameters; however, effects on IL-6 are inconsistent and often not statistically significant [50, 51]. These observations suggest that chromium may dampen inflammatory transcription, although the extent to which this reflects direct versus indirect effects likely depends on formulation and experimental context. A key mechanistic link between chromium-related metabolic and anti-inflammatory effects is AMPK. Beyond its role in substrate metabolism, AMPK suppresses NF-κB transcriptional activity through deacetylation-dependent and stress-limiting mechanisms, thereby coupling energetic adaptation to inflammatory control [47, 48]. In parallel, Nrf2 activation reduces ROS-driven inflammatory amplification, further limiting NF-κB activation. Thus, suppression of inflammatory signaling in chromium-treated models is most plausibly interpreted within the broader ATP synthase–AMPK–Nrf2 network rather than as an isolated anti-inflammatory action. Overall, the available data support a model in which CrIII may attenuate obesity-associated inflammation by improving energy sensing, enhancing antioxidant defenses, and thereby secondarily limiting NF-κB-driven transcription. This integrated view is more consistent with the complexity of obesity-related metabolic dysfunction than a narrow insulin-centered interpretation and helps explain why the metabolic and inflammatory effects of chromium often appear mechanistically linked rather than independent. Importantly, the chromium(III) doses and concentrations used in experimental models (Table 2) including micromolar ranges in vitro (e.g., ~ 25–200 µM) and mg/kg body weight levels in vivo (e.g., ~ 0.3–10 mg/kg/day depending on formulation) substantially exceed baseline levels present in standard culture media and laboratory diets, where chromium exists only at trace levels. Therefore, the observed metabolic and anti-inflammatory effects should be interpreted within a supranutritional, dose-dependent framework rather than reflecting physiological chromium exposure.

**Table 2:** Mechanistic effects of chromium(III) on energy metabolism, redox balance, inflammation, insulin signaling, and lipid metabolism in experimental models

Domain	Mechanistic Target	Model	Chromium Form	Dose / Concentration	Key Evidence	Metabolic Outcome	References
Energy (AMPK)	Mitochondrial ATP synthase (proposed)	HepG2; C2C12; HeLa; db/db mice	CrCl₃; Cr³⁺–NTA-AC (probe complex); (OM2, ~ 2% Cr)	In vitro: 50–200 µM CrCl₃; ~50 µM Cr³⁺–NTA-AC; 25–50 µM OM2; In vivo: 10 mg/kg/day	CrIII interacts with mitochondrial ATP synthase and increases the AMP/ATP ratio, leading to AMPK activation; OM2 activates AMPK–PGC-1α signaling and mitochondrial biogenesis.	Associated with improved mitochondrial function and energy metabolism	[23, 40]
Energy (AMPK–PGC-1α)	AMPK–PGC-1α axis	3T3-L1 adipocytes, C2C12 myotubes	Oligomannuronate–CrIII complexes (OM2, ~ 2% Cr; OM4); Chromium picolinate (CrPic)	OM2/OM4: 25–50 µM (optimal range); CrPic: ~100 nM	OM2 activates AMPK–PGC-1α signaling, increasing mitochondrial biogenesis and fatty acid oxidation; CrPic induces AMPK phosphorylation in an AMPK-dependent manner	Enhanced mitochondrial function, reduced lipid accumulation, and improved insulin responsiveness	[39, 40]
Redox (Nrf2/HO-1)	Keap1–Nrf2 signaling	HFD-fed rats; STZ + HFD diabetic rats	CrHis; CrPic	CrHis: 110 µg/kg, CrPic/CrHis: ~8 µg Cr/day	Increased Nrf2 and HO-1 expression and reduced lipid peroxidation (HNE), with concurrent suppression of NF-κB activation	Associated with reduced oxidative stress and attenuation of inflammation-related metabolic disturbances	[25, 26]
Redox–Inflammation	Oxidative stress–inflammation coupling (GSH/GSSG, IL-6, TNF-α, COX-2)	HFD rats	CrPic; Cr-NPs; Cr-Met	0.3 mg/kg BW	Chromium supplementation modulates redox and inflammatory markers, including increased GSH/GSSG ratio and reduced IL-6 and COX-2 expression, although some forms increase pro-inflammatory cytokines (IL-6, TNF-α) depending on formulation	Associated with modulation of oxidative stress and inflammatory responses in obesity-related conditions	[27, 114]
Inflammation (NF-κB)	NF-κB signaling (indirect modulation)	HFD/STZ-induced diabetic rats	CrPic; CrHis	CrPic/CrHis: ~8 µg Cr/day,	CrPic and CrHis decrease NF-κB p65 expression and increase IκBα levels, indicating suppression of inflammatory signaling	Associated with reduced inflammation and improved metabolic disturbances in diabetic conditions	[26, 49]
Insulin signaling	IRS-1 / PI3K / Akt /GLUT4 pathway	C2C12 skeletal muscle cells; 3T3-L1	OM2, OM4; Cr-Mal;	OM2/OM4: ~50 µM; Cr-Mal: 5–20 µg Cr/mL	Chromium complexes activate IRS-1/PI3K/Akt signaling and increase GLUT4 expression while reducing inhibitory IRS-1 phosphorylation	Enhanced glucose uptake and improved insulin sensitivity at the cellular level	[41, 84]
Obesity / Lipid metabolism	AMPK–PGC-1α axis; PPARα; ATGL-mediated lipolysis; hepatic lipid metabolism regulators (COX-2, HIF-1α)	3T3-L1 adipocytes; HFD-fed rats	OM2, ~ 2% Cr; OM4; CrPic; Cr-NPs	OM2/OM4: 25–50 µM; Cr (in vivo): CrCl₃: 0.3 mg/kg BW	OM2 activates AMPK–PGC-1α signaling, increasing fatty acid oxidation and reducing triglyceride accumulation; chromium supplementation modulates hepatic lipid metabolism via increased PPARα and reduced COX-2 and HIF-1α	Reduced lipid accumulation and improved fatty acid metabolism in obesity-related models	[27, 40]

*AMPK* AMP-activated protein kinase, *PGC-1α* peroxisome proliferator-activated receptor gamma coactivator-1 alpha, *Nrf2* nuclear factor erythroid 2–related factor 2, *HO-1* heme oxygenase-1, *NF-κB* nuclear factor kappa B, *IκBα* inhibitor of κB alpha, *IRS-1* insulin receptor substrate-1, *PI3K* phosphoinositide 3-kinase, *Akt* protein kinase B, *GLUT4* glucose transporter type 4, *JNK* c-Jun N-terminal kinase, *PPARα* peroxisome proliferator-activated receptor alpha, *ATGL* adipose triglyceride lipase, *COX-2* cyclooxygenase-2, *HIF-1α* hypoxia-inducible factor 1 alpha, *GSH* reduced glutathione, *GSSG* oxidized glutathione, *IL-6* interleukin-6, *TNF-α* tumor necrosis factor-alpha.

Chromium forms include CrCl₃, CrPic, CrHis, Cr-Mal, Cr(D-Phe)₃, Cr-NPs, and oligomannuronate–CrIII complexes (OM2/OM4). In vitro concentrations are expressed as µM or µg/mL, whereas in vivo doses are given as µg/kg or mg/kg body weight; fixed daily doses indicate non–body weight-adjusted administration. HFD indicates high-fat diet and STZ indicates streptozotocin-induced diabetes. Effects are based on experimental models and represent mechanistic associations

### AMPK↔Nrf2 Cross-talk: Why Dual Activation Matters

Perhaps more importantly, a novel metabolic axis that links AMPK and Nrf2 has recently been described as an interconnected hub overseeing energy sensing and redox balance. The activation of Nrf2 by AMPK occurs directly through phosphorylation-mediated nuclear entry and indirectly via inhibition of GSK3, which would otherwise target Nrf2 for degradation [6, 52]. Furthermore, AMPK upregulates the transcription of a subset of Nrf2 target genes (including HO-1) by negatively regulating the transcriptional repressor Bach1 and by reducing endoplasmic reticulum stress [53, 54]. Conversely, Nrf2-regulated oxidative stress programs redress mitochondrial redox and NADPH and glutathione pools, restrict ROS generation at the source while preserving AMPK sensitivity, and curb NF-κB–mediated inflammatory signaling [43, 45, 55]. Functional cross-talk exists between these pathways, as revealed in inflammatory models showing that AMPK activators like berberine require an intact Nrf2 signaling pathway to fully suppress oxidative stress and inflammatory gene expression [56]. In this “beyond insulin” chromium model, the bidirectional AMPK↔Nrf2 cross-talk is also highly relevant. CrIII-triggered AMPK activation, induced by mitochondrial ATP synthase disruption, might subsequently reinforce Nrf2/HO-1 signaling, which is in turn controlled by Nrf2-dependent redox balancing as a feedback regulator of the AMPK response and NF-κB deactivation. Such orchestrated coordination of energy sensing and antioxidant defense would be particularly relevant in obesity, where lipid overload, mitochondrial stress, and chronic inflammation coalesce to undermine metabolic flexibility. Altogether, the available evidence portrays CrIII as a context-dependent regulator of the redox–energy cross-talk, able to recalibrate the tone of oxidative stress and inflammation via the Nrf2/HO-1 axis, concomitant with interfacing with AMPK-mediated metabolic control. Rather than serving as a mere insulin adjunct, CrIII may reset the set point of interconnected redox and energy-sensing networks, thereby providing a mechanistic basis for its pleiotropic roles in obesity and other cardiometabolic disorders.

### Chromium(III) and Inflammatory Tone: Restraining NF-κB in Obesity

#### NF-κB as A Driver of Metaflammation

Obesity is now widely recognized as a state of chronic, low-grade inflammation termed metaflammation that mechanistically links nutrient excess to insulin resistance, tissue remodeling, and cardiometabolic disease. At the center of this process lies NF-κB, a master transcriptional regulator that integrates diverse metabolic stress signals into a sustained pro-inflammatory gene expression program [57–59]. In metabolically active tissues such as white adipose tissue, liver, and skeletal muscle, NF-κB activation is not driven by a single upstream stimulus but rather by the convergence of multiple stressors, including lipotoxicity induced by saturated fatty acids, oxidative stress, endotoxin exposure, and endoplasmic reticulum (ER) stress [60–62]. ER stress, through activation of PERK, IRE1, and ATF6 signaling pathways, further amplifies NF-κB signaling in conjunction with stress kinases such as JNK and p38 MAPK, thereby enhancing transcription of inflammatory genes [61, 63].

Persistent NF-κB activation disrupts insulin signaling through multiple coordinated mechanisms. It promotes serine phosphorylation of insulin receptor substrate-1 (IRS-1) via IKKβ and JNK, impairing downstream PI3K/Akt signaling and reducing GLUT4-mediated glucose uptake in peripheral tissues [64, 65]. At the same time, NF-κB induces negative regulators of insulin signaling, including suppressor of cytokine signaling-3 (SOCS3) and protein tyrosine phosphatase 1B (PTP1B), further attenuating insulin receptor function [57, 58]. In addition, NF-κB activation enhances lipogenesis and promotes lipotoxic stress, further impairing insulin action [66]. These processes collectively lead to reduced insulin sensitivity, increased hepatic glucose production, and diminished glucose uptake in skeletal muscle and adipose tissue. Importantly, NF-κB signaling operates within tissue-specific immunometabolic circuits that amplify metaflammation. In adipose tissue, NF-κB activation promotes the expression of chemokines such as MCP-1, facilitating macrophage recruitment and polarization toward a pro-inflammatory M1 phenotype, which sustains cytokine production and local inflammation [62, 67]. In the liver, even modest NF-κB activation is sufficient to induce both local and systemic insulin resistance by increasing IL-6, TNF-α, and acute-phase proteins such as C-reactive protein [59, 68]. In skeletal muscle, lipid overload and exposure to saturated fatty acids activate IKKβ/NF-κB signaling, impairing insulin-stimulated glucose uptake and contributing to systemic metabolic dysfunction [65].

A defining feature of obesity-associated metaflammation is the presence of reinforcing feedback loops. Pro-inflammatory cytokines, free fatty acids, and reactive oxygen species generated downstream of NF-κB activation further stimulate upstream signaling pathways, including Toll-like receptor 4 (TLR4) and ER stress responses, thereby sustaining NF-κB activation in a feed-forward manner [67, 69, 70]. Additionally, emerging evidence indicates that non-canonical regulators, such as REDD1, can activate NF-κB independently of classical IKK signaling, further strengthening inflammatory signaling and metabolic dysfunction [71]. Systems biology and multi-omics analyses consistently identify NF-κB as a central hub in the inflammatory networks underlying insulin resistance, underscoring its integrative role across metabolic tissues [66]. Within this framework, the potential role of chromium(III) may be more appropriately interpreted not as a direct insulin sensitizer, but as a context-dependent modulator of inflammatory tone. By influencing redox balance, cellular stress responses, or upstream signaling pathways, CrIII could theoretically attenuate NF-κB activation and thereby indirectly improve metabolic function. However, the available evidence remains heterogeneous and does not consistently demonstrate robust or clinically meaningful anti-inflammatory effects of chromium across experimental and clinical studies. Therefore, while modulation of the NF-κB axis represents a plausible mechanistic pathway, it should be considered within a broader, context-dependent network of metabolic regulation rather than as a singular explanatory mechanism, and only to the extent that CrIII actually has meaningful biological effects.

### How CrIII may suppress NF-κB: Indirect but convergent mechanisms

Direct inhibition of NF-κB by CrIII has not been conclusively demonstrated. Instead, the available evidence supports a model in which CrIII modulates NF-κB activity indirectly by attenuating its upstream drivers, particularly those linked to cellular energy imbalance and oxidative stress. This perspective aligns with the emerging immunometabolic framework, where inflammatory signaling is not regulated by isolated pathways but by the integration of energy status, redox balance, and stress signaling networks.

One major axis through which CrIII may influence inflammatory tone is the mitochondrial energy-sensing pathway. Recent work has identified mitochondrial ATP synthase as a potential molecular target of CrIII, in which binding to the β subunit leads to a modest reduction in ATP production, an increase in the AMP/ATP ratio, and subsequent activation of AMP-activated protein kinase (AMPK) [23, 29]. However, this mechanism is primarily supported by in vitro and experimental models, and its physiological relevance in humans remains uncertain. Beyond this ATP synthase–linked mechanism, chromium picolinate has been reported to activate AMPK in L6 skeletal muscle cells, with siRNA-mediated AMPK depletion abolishing its effects on GLUT4 translocation and glucose transport [39]; however, as this study employed chromium picolinate rather than inorganic CrCl₃, the specific contribution of the chromium ion versus the picolinate ligand cannot be clearly distinguished. Similarly, oligomannuronate–CrIII complexes have been shown to activate the AMPK/PGC-1α axis in C2C12 myotubes and 3T3-L1 adipocytes [40, 41]; notably, equivalent concentrations of inorganic CrCl₃ did not reproduce these effects, indicating that the observed activity may be largely attributable to the ligand rather than chromium itself. In addition, co-supplementation studies (e.g., chromium with magnesium) report improvements in metabolic and inflammatory parameters, although these effects are not consistently observed with chromium alone, further limiting attribution of these outcomes specifically to chromium [72]. A second potential mechanism involves modulation of redox homeostasis through activation of the Nrf2 pathway. Oxidative stress is a key upstream driver of NF-κB activation, and some studies report that chromium supplementation is associated with reductions in lipid peroxidation markers and increases in antioxidant capacity [72, 73]. Experimental data also suggest activation of the Keap1/Nrf2/HO-1 pathway in response to certain chromium-containing compounds [43, 74]. However, these findings are heterogeneous, often formulation-dependent, and do not establish a consistent or direct mechanistic effect of chromium itself. Given the well-established cross-talk between Nrf2 and NF-κB signaling, improvements in redox balance may contribute to reduced inflammatory signaling; however, such effects are likely indirect and context-dependent rather than evidence of a defined chromium-specific mechanism. These limitations are particularly relevant for chromium complexes containing biologically active ligands. A substantial body of evidence indicates that different CrIII complexes exhibit ligand-dependent biological effects, with ligands such as amino acids, organic acids, or polyphenols possessing intrinsic metabolic and antioxidant properties that may significantly influence observed outcomes [75–77]. In some cases, ligand-specific contributions may dominate both efficacy and safety profiles, complicating interpretation of chromium’s independent role [14, 77]. Therefore, in the absence of appropriate chromium-only and ligand-only controls, attribution of biological effects specifically to chromium is not justified and such findings should be interpreted with caution.

Downstream of these upstream effects, several experimental studies report improvements in glucose metabolism and insulin signaling in response to chromium supplementation. In insulin-resistant and obese models, chromium complexes have been associated with improved glucose tolerance and increased Akt phosphorylation, alongside activation of AMPK-related pathways; however, evidence for consistent effects on inflammatory cytokines and oxidative stress markers remains limited and model-dependent [41, 73, 78, 79]. These improvements are likely not the result of direct NF-κB inhibition, but rather reflect a generalized dampening of pro-inflammatory output due to normalization of cellular energy and redox states. In this context, reduced activation of stress kinases such as JNK and decreased serine phosphorylation of IRS-1 further contribute to improved insulin signaling.

A key implication of this model is that NF-κB activation in obesity is maintained by multiple overlapping and mutually reinforcing upstream signals, including energy excess, lipotoxicity, oxidative stress, and ER stress. Interventions targeting a single pathway, such as cytokine signaling or insulin sensitivity, may therefore be insufficient to reverse chronic metaflammation fully. In contrast, CrIII appears to act on at least two major upstream nodes simultaneously: energy sensing via AMPK and redox regulation via Nrf2. By attenuating both energetic and oxidative stress inputs, CrIII may reduce the convergent signaling required to sustain NF-κB activation.

This dual modulation provides a mechanistic basis for how chromium supplementation can produce coordinated, albeit modest, improvements across multiple domains, including insulin sensitivity, oxidative stress markers, and inflammatory status. Within this framework, the classical view of chromium as an insulin sensitizer is reframed: improvements in insulin signaling may arise secondarily from the attenuation of NF-κB-driven metaflammation and restoration of cellular stress resilience [25, 29]. Accordingly, CrIII may be better conceptualized not as a direct inhibitor of inflammatory pathways, but as a context-dependent modulator of inflammatory tone, acting through integrated energy–redox mechanisms. This systems-level perspective situates chromium biology within the broader landscape of immunometabolism, although the clinical relevance of these proposed mechanisms remains to be established in future studies.

### Integrating Classical Insulin Signaling with A “Beyond Insulin” Chromium(III) Model

While the overall framework of this review extends beyond insulin signaling, the classical interactions between chromium(III) and insulin pathways remain an important component of its biological profile. A substantial body of experimental and clinical literature indicates that CrIII can enhance insulin receptor–proximal events, including increased insulin receptor phosphorylation, improved IRS-1 signaling, and enhanced downstream PI3K/Akt pathway activation. These changes are frequently accompanied by increased GLUT4 translocation and glucose uptake in insulin-sensitive tissues such as skeletal muscle and adipose tissue. However, the magnitude, consistency, and clinical relevance of these effects remain variable across studies and, in many cases, modest, highlighting the need for a broader interpretative framework.

When considered in isolation, these findings have historically supported the concept of chromium as an “insulin sensitizer” or a modulator of insulin signaling. However, as discussed throughout this review, such a unidimensional interpretation is insufficient given the breadth of experimental evidence now supporting involvement of non-insulin pathways. Increasing evidence suggests that the apparent enhancement of insulin signaling by CrIII may not always represent a primary, receptor-specific action, but rather may emerge secondarily from improvements in the cellular environment in which insulin signaling operates. In particular, CrIII appears to influence two major upstream determinants of insulin responsiveness: cellular energy status and redox balance. Through modulation of mitochondrial function and activation of AMP-activated protein kinase (AMPK), CrIII can improve energy sensing, promote fatty acid oxidation, and reduce intracellular lipid accumulation factors known to impair insulin signaling through lipotoxic mechanisms. In parallel, by enhancing antioxidant defenses, including activation of the Nrf2/HO-1 pathway, CrIII may reduce oxidative stress and limit the activation of stress kinases such as JNK and IKKβ, which otherwise promote inhibitory serine phosphorylation of IRS-1 and disrupt insulin receptor signaling. Importantly, these energy and redox effects converge on inflammatory pathways, particularly NF-κB signaling, which plays a central role in metaflammation and insulin resistance. By attenuating upstream drivers of NF-κB activation such as reactive oxygen species, ER stress, and lipid overload CrIII may indirectly reduce pro-inflammatory cytokine production (e.g., TNF-α, IL-6) and relieve inflammation-mediated inhibition of insulin signaling. In this context, improvements in insulin sensitivity may reflect the normalization of an adverse immunometabolic milieu rather than direct potentiation of insulin receptor function. This integrated perspective suggests that insulin sensitization should be viewed as one component within a broader, systems-level response to CrIII, rather than its primary or exclusive mode of action. The effects of CrIII appear to arise from coordinated modulation of four interconnected pathways: energy metabolism (AMPK), redox homeostasis (Nrf2), inflammatory signaling (NF-κB), and classical insulin signaling (IR→IRS-1→PI3K→Akt→GLUT4) the last of which is not the primary entry point but remains an integral component of the network, itself regulated upstream by the other three axes. Such a model also helps explain why chromium supplementation may yield modest or inconsistent effects on isolated glycemic endpoints while still exerting measurable influences on oxidative stress markers, inflammatory tone, or mitochondrial function. Taken together, these observations support a “beyond insulin” model of chromium biology, in which CrIII acts as a context-dependent modulator of metabolic resilience. Rather than functioning solely as an insulin sensitizer, chromium may enhance the capacity of cells and tissues to respond to metabolic stress, thereby indirectly improving insulin signaling when and only when underlying energetic, oxidative, or inflammatory disturbances are present. This framework reconciles classical findings with more recent mechanistic insights and provides a more comprehensive explanation for the diverse and sometimes inconsistent biological effects attributed to chromium in obesity and cardiometabolic disease.

### Direct Effects on the Insulin Signaling Machinery

A substantial body of experimental evidence supports a classical role for chromium(III) as an amplifier of the insulin signaling cascade, spanning receptor-proximal and post-receptor events across multiple metabolic tissues. In both cell-based systems and in vivo models, CrIII has been shown to enhance key nodes of the canonical IR/IRS-1/PI3K/Akt/GLUT4 pathway, although the magnitude and consistency of these effects vary across experimental contexts. In skeletal muscle of obese diabetic KK/HlJ mice, chromium supplementation via chromium-enriched milk restored IRS-1 protein levels and tyrosine phosphorylation, increased PI3K p85α subunit expression, and enhanced Akt activity and GLUT4 abundance. Improvements in glycemia, hyperinsulinemia, and lipid profiles accompanied these molecular changes [8]. Notably, chromium supplementation also reduced JNK activation, decreased inhibitory IRS-1 Ser307 phosphorylation, and limited IRS-1 ubiquitination, thereby restoring insulin responsiveness at multiple regulatory checkpoints within the signaling cascade [80, 81]. Similarly, in obese insulin-resistant JCR: LA-cp rats, chromium picolinate increased insulin-stimulated IRS-1 phosphorylation and PI3K activity in skeletal muscle while reducing both the expression and activity of protein tyrosine phosphatase 1B (PTP1B), a key negative regulator of insulin receptor signaling [82]. This effect likely prolongs receptor signaling and enhances downstream metabolic responses [83].

Complementary evidence from in vitro models further supports direct modulatory effects of chromium on insulin signaling components. In adipocytes and myotubes, including 3T3-L1 and L6 cells, chromium complexes such as chromium picolinate, chromium malate, and oligomannuronate–CrIII have been shown to increase the expression and phosphorylation of IRS-1, PI3K, Akt, and PPARγ, while upregulating GLUT4 and enhancing glucose uptake. These effects are often accompanied by reduced inhibitory serine phosphorylation of IRS-1, suggesting improved signal fidelity [41, 84]. In C2C12 myotubes, oligomannuronate–CrIII complexes enhanced insulin receptor expression and downstream PI3K/Akt signaling, further supporting a role for chromium in amplifying insulin action at multiple levels of the cascade [41]. Additionally, cellular chromium has been shown to enhance insulin receptor kinase activity and receptor autophosphorylation, although these effects do not appear to be mediated through direct inhibition of PTP1B, indicating alternative regulatory mechanisms at the receptor level [85, 86].

Beyond these direct signaling effects, increasing evidence indicates that CrIII also modulates the cellular context in which insulin signaling occurs. In particular, chromium compounds have been shown to activate AMP-activated protein kinase (AMPK), improve mitochondrial function, and influence membrane organization and cytoskeletal dynamics involved in GLUT4 vesicle trafficking. For example, in L6 myotubes, chromium picolinate prevented hyperinsulinemia-induced membrane cholesterol accumulation and cortical F-actin disruption, thereby restoring GLUT4 translocation independently of major changes in proximal IR/IRS-1/Akt phosphorylation. Importantly, these effects were abolished upon AMPK inhibition, highlighting a critical role for energy-sensing pathways in mediating chromium’s insulin-sensitizing actions [39]; however, it should be noted that this study employed chromium picolinate rather than inorganic CrCl₃, and the specific contribution of the chromium ion versus the picolinate ligand was not independently assessed. Additional studies indicate that chromium may also engage alternative signaling nodes, including p38 MAPK and PPARγ, suggesting that its effects extend beyond the canonical insulin cascade [84, 87]. These findings mainly arise from experimental models and do not establish a clear role for chromium in humans. In addition, bioactive ligands in chromium complexes may independently drive the observed effects, limiting attribution to chromium specifically [14].

Oxidative stress and inflammation are well-established inhibitors of insulin signaling, acting through mechanisms such as IRS-1 serine phosphorylation, PI3K/Akt uncoupling, and impaired GLUT4 translocation. In this context, chromium supplementation has been consistently associated with reductions in oxidative stress markers and improvements in antioxidant defenses in both experimental models and clinical studies [82, 88]. These effects are linked to activation of the Nrf2/HO-1 pathway and attenuation of NF-κB–mediated inflammation, resulting in decreased levels of pro-inflammatory cytokines such as TNF-α and IL-6. Importantly, the restoration of insulin signaling observed in these models often parallels improvements in redox and inflammatory status, suggesting that the insulin-sensitizing effects of CrIII may, at least in part, arise from correcting upstream cellular stressors rather than direct receptor-level modulation alone [87, 88]. Overall, these findings support a unified model in which CrIII potentiates insulin signaling through both direct and indirect mechanisms. At the canonical level, CrIII enhances the IR/IRS-1/PI3K/Akt/GLUT4 cascade and reduces the activity of key negative regulators, such as PTP1B and JNK, and inhibits inhibitory IRS-1 serine phosphorylation. At the same time, CrIII improves the broader cellular environment by modulating energy status (AMPK), membrane dynamics, oxidative stress (Nrf2/HO-1), and inflammatory tone (NF-κB). Within this integrated framework, enhancement of insulin signaling emerges not merely as a direct receptor-level effect, but as a downstream consequence of improved cellular homeostasis.

This perspective reconciles decades of chromium research focused primarily on insulin signaling with more recent insights into its roles in energy metabolism, redox regulation, and immunometabolic control. It also provides a mechanistic explanation for the context-dependent, often modest metabolic effects observed in clinical studies, suggesting that chromium’s benefits may be better understood as the cumulative outcome of coordinated modulation across multiple interconnected pathways rather than as isolated effects on any single signaling node.

### Translational Evidence: What do Human Data Say, and Why are Results Heterogeneous?

Many years of experimental and mechanistic research notwithstanding, translating CrIII supplementation into reliable clinical benefit in obesity and diabetes has been problematic. In randomized controlled trials (RCTs) and meta-analyses, chromium supplementation produces statistically significant but modest mean effects that are highly variable across studies. The available clinical data are insufficient to validate or refute any specific mechanism of action.

Recent quantitative syntheses consistently indicate small but measurable metabolic effects following chromium supplementation, despite substantial heterogeneity across populations and trial designs. A 2025 meta-analysis in overweight and obese adults demonstrated modest reductions in fasting insulin, HOMA-IR, and body weight, whereas effects on BMI, waist circumference, triglycerides, and most lipid parameters were not statistically significant, and liver enzyme responses were variable [89]. Previous meta-analyses in overweight and obese populations similarly reported statistically significant but clinically modest decreases in body weight (approximately 0.5–0.75 kg), BMI, and body fat mass, while emphasizing the limited clinical relevance of these effects and substantial inter-individual variability [18, 90]. In type 2 diabetes, a pooled analysis also found modest overall improvements in fasting glucose, HbA1c, insulin, and HOMA-IR, again with very high I²% and by formulation-specific effects, as indicated for chromium chloride and chromium picolinate [17, 91]. There are also small lipid effects: chromium supplementation decreases total cholesterol and triglycerides and increases HDL cholesterol in T2DM with modest effect sizes [22]. Meta-analyses indicate that chromium supplementation may reduce some inflammatory biomarkers, including hs-CRP and TNF-α [92], although effects on IL-6 were not significant; similarly, improvements in MDA and TAC have been reported [73]; but these were based on a limited number of studies with substantial heterogeneity, and no significant effects were observed for CAT, GPx, GSH, SOD, TAS, or TBARS. Overall, these findings are mixed and characterized by substantial heterogeneity and limited sample sizes. General clinical reviews consequently conclude that, although there are statistically significant signals, the overall clinical effect is marginal and inconsistent, leading to a lack of consistent recommendation for chromium supplementation in the management of obesity or diabetes in unselected populations [14]. Nevertheless, these findings are based on population averages, not on the absence of physiologically relevant responses in subgroups. Importantly, this heterogeneity closely mirrors the historical pattern first described by Mertz and Anderson [11]. In many early chromium supplementation studies, beneficial effects were preferentially observed in subjects exposed to metabolic challenges, including oral glucose tolerance tests, low chromium intake, diabetes, or nutritional stress, whereas healthy and metabolically stable individuals often exhibited minimal responses [11–13]. More recent animal and human studies similarly suggest that chromium responsiveness is amplified in the presence of physiological perturbations such as insulin resistance, impaired glucose tolerance, obesity, oxidative stress, or heat stress [93–95]. Collectively, these findings support the interpretation that chromium(III) acts primarily as a context-dependent modulator capable of partially restoring disrupted metabolic homeostasis rather than exerting robust metabolic effects in healthy physiological states.

Importantly, a pronounced discrepancy exists between chromium doses used in experimental models and those applied in human studies. In rodent models, chromium is often administered at pharmacological levels (e.g., ~ 0.3–1 mg/kg/day), which are substantially higher, on a body weight basis, than doses used in human supplementation trials [27, 96, 97]. In contrast, clinical studies in humans typically employ doses in the range of 200–1000 µg/day, which are closer to physiological exposure levels [16, 22, 73, 91]. This translational gap suggests that many mechanistic effects observed in experimental systems may arise under pharmacological rather than physiological conditions, which may partly explain the limited and inconsistent clinical efficacy reported in human studies [14, 96].

### Why are the results heterogeneous? Limitations of clinical evidence and cautious mechanistic interpretation

This heterogeneity does not support any specific molecular mechanism, and should not be interpreted as evidence for or against a particular pathway, including the proposed ATP synthase–AMPK/Nrf2/NF-κB model [14, 98]. Rather than supporting a defined molecular mechanism, the clinical evidence is characterized by inconsistency and limited effect sizes, as also emphasized in broader evaluations of chromium supplementation [14]. Some meta-analyses report nominally greater signals in individuals with overt insulin resistance or type 2 diabetes; however, these subgroup findings should be interpreted with particular caution given the methodological limitations described above, including substantial heterogeneity, small sample sizes, and potential bias [16–18, 91]. In NAFLD cohorts, chromium picolinate has been reported to improve insulin indices, triglycerides, and certain inflammatory markers without marked changes in glycaemia or liver fat content, suggesting that responses may depend on underlying metabolic and inflammatory status rather than simple adiposity [99]. Similarly, co-supplementation of chromium and magnesium in impaired glucose tolerance (IGT) was associated with improvements in insulin resistance and inflammatory markers; however, chromium alone did not produce significant within-group reductions in fasting glucose or fasting insulin, and the independent contribution of chromium cannot be separated from that of magnesium in this study design [72]. Importantly, the heterogeneity observed across clinical studies is likely driven by multiple interacting factors, including differences in chromium formulation, dosage, intervention duration, baseline metabolic status, and study design. Moreover, the modest and inconsistent effects observed in human studies contrast with more robust responses reported in experimental models, where substantially higher, pharmacological doses are often used, suggesting a potential mismatch between experimental and clinical dosing conditions. In addition, subgroup analyses within meta-analyses, particularly when based on a limited number of studies, are highly susceptible to false-positive findings driven by selection criteria and methodological variability. Differences in inclusion criteria, baseline metabolic status, and formulation-specific effects may largely explain the transition from null to statistically significant but clinically marginal outcomes across meta-analyses. Consequently, such findings should be interpreted with caution and should not be considered mechanistic evidence. This interpretation is also consistent with the classical Mertz–Anderson [11] ( framework, which proposed that chromium supplementation exerts its most reproducible biological effects under conditions of metabolic challenge or nutritional inadequacy rather than in healthy individuals with preserved metabolic homeostasis.

### What Clinical Trials Should Measure Next: Mechanism-Linked Biomarkers

Most human chromium RCTs focus on distal endpoints such as glycaemia, body weight, and lipid profiles, with minimal mechanistic characterization. Only a minority of trials evaluate inflammatory or oxidative markers, and among those that do, results are mixed; a small number of studies report reductions in certain markers such as hs-CRP, TNF-α, MDA, and TAC, but these findings are based on a limited number of studies with substantial heterogeneity, and no significant effects were observed for the majority of biomarkers assessed, including IL-6, CAT, GPx, GSH, SOD, TAS, and TBARS. However, direct assessments of AMPK activation, Nrf2/HO-1 signaling, or NF-κB activity are almost entirely absent. Rigorous testing of the ATP synthase/AMPK/Nrf2/NF-κB hypothesis in humans will require incorporation of mechanism-linked biomarkers in future studies involving obesity, NAFLD, and T2DM. These biomarkers should include indicators of cellular energy sensing such as phosphorylated AMPK, ACC phosphorylation, PGC-1α levels, and mitochondrial biogenesis markers measured in peripheral blood mononuclear cells (PBMCs) or tissue biopsies; measures of redox defense including HO-1, NQO1, and Glutamate–cysteine ligase catalytic subunit (GCLC) expression, as well as plasma or erythrocyte concentrations of MDA, F₂-isoprostanes, TAC, and GSH/GSSG ratios; indices of inflammatory tone such as NF-κB target gene panels, hs-CRP, IL-6, TNF-α, MCP-1, and key adipokines; and readouts of mitochondrial or ectopic lipid status, including metabolomics-based signatures, acylcarnitine profiles, and magnetic resonance imaging–proton density fat fraction (MRI-PDFF) assessments of liver fat. Equally important is stratifying participants by metabolic phenotype, degree of inflammatory burden, NAFLD status, and, where possible, validated measures of chromium exposure or tissue incorporation.

Human trials of chromium supplementation in obesity and diabetes reveal small average benefits, if any, with striking heterogeneity across populations, formulations, doses, and durations. This variability cannot be interpreted as evidence supporting a specific molecular mechanism. Rather, the modest and inconsistent clinical findings indicate that current human data are insufficient to validate any mechanistic framework, including the proposed ATP synthase–AMPK/Nrf2/NF-κB model. Alternative explanations include minimal true biological effect, insufficient dosing relative to experimental models, and substantial methodological heterogeneity across studies. Moving the field forward will require mechanism-anchored, phenotype-stratified RCTs that test whether modulation of these pathways mediates clinical benefit, thereby defining whether chromium has a defensible role as a context-specific metabolic modulator rather than a general-purpose “weight-loss” or “insulin” supplement.

### Safety, Essentiality, and Regulatory-Scientific Uncertainty

Any translation of the valency-content of chromium in obesity and cardiometabolic disease must unequivocally address the differentiation between CrIII and chromium(VI) (CrVI), as well as tackle an emerging regulatory, scientific ‘battle’ regarding chromium essentiality, safety margins, and clinical position. Recent high-level reviews have moved the field from a simplistic view of CrIII as an “essential trace element” towards a more prudent, context-dependent view of CrIII biology.

### CrIII Versus CrVI: Distinct Toxicology, Distinct Regulatory Framing

CrVI and CrIII are fundamentally different in their toxicology profiles and regulatory handling. CrVI, to which humans are most commonly exposed occupationally and environmentally, is well established as a Group 1 human carcinogen with high cellular permeability that causes oxidative DNA damage through genotoxic mechanisms [10, 100, 101]. CrIII, on the other hand, is the most prevalent dietary and supplemental form of the trace element and has been classically related to carbohydrate, lipid, and protein metabolism. Nevertheless, the reduced inherent toxicity of CrIII does not mean there is no risk. Although CrIII is generally less bioavailable and genotoxic than CrVI, a number of comprehensive reviews highlight that some CrIII complexes in particular (e.g., chromium picolinate and nanoparticulate forms) can have cytotoxic or genotoxic effects at high doses or after prolonged exposures, especially if contaminated with CrVI, or when intracellular redox cycling is enhanced [10, 100–102]. These distinctions underscore why regulatory agencies assess CrIII and CrVI through fundamentally different lenses, and why translational discussions must avoid conflating their safety profiles.

### Essentiality and Dietary Recommendations: A Shifting Consensus

Chromium, as an essential nutrient, has been the subject of heated debate. The scoping review on NNR 2023 found that there is little evidence for reliable biomarkers of chromium status, that deficiency syndromes have not been described in healthy individuals, and that the available evidence was insufficient to establish DRV values for chromium intake [15]. This stance aligns with previous statements issued by the European Food Safety Authority (EFSA), which has not set an Adequate Intake for chromium because there is little evidence of benefit in healthy populations. These findings, however, are at odds with historical recommendations in the US and Canada, which were largely derived from case reports (e.g., long-term total parenteral nutrition) or early supplementation studies proposing effects on insulin and lipid metabolism [103, 104]. Narrative and toxicological reviews have increasingly characterized CrIII as pharmacologically active rather than nutritionally essential, suggesting that supplementation is not justified for individuals consuming varied diets [14, 105]. While some authors continue to support an essentialist view, these arguments are now widely regarded as context-specific and insufficient to support population-wide intake recommendations.

### Positioning CrIII in Obesity and Metabolic Disease

This is of regulatory significance for how CrIII should be defined regarding obesity and metabolic disorders. Multiple recent reviews directly recommend against “routine” chromium supplementation for weight loss or metabolic health, due to small and possibly unreliable clinical effects with unresolved mechanisms of action, as well as incomplete longer-term safety data, especially at high doses or in specialized forms [14, 16, 105]. The NNR scoping review also highlights that doses of chromium required to elicit metabolic effects in obesity and diabetes trials are much greater than those achievable through a normal diet, supporting the view that CrIII is a pharmacological modulator rather than an essential dietary element [15]. This distinction requires further mechanistic clarification. The effects of CrIII are unlikely to be confined to a single pathway but instead reflect an integrated network involving insulin signaling, mitochondrial energy sensing (ATP synthase–AMPK), redox regulation (Nrf2/HO-1), and inflammatory signaling (NF-κB). Importantly, these pathways are highly interconnected: AMPK activation can enhance insulin sensitivity and modulate NF-κB–mediated inflammation; Nrf2 signaling influences cellular redox status, which in turn regulates both insulin signaling and inflammatory responses; and NF-κB activation can impair insulin signaling through inflammatory feedback loops. Within this framework, the context-dependent effects of CrIII are better interpreted as modulating this interconnected signaling network rather than as a mechanism independent of insulin pathways. Consequently, its biological effects may depend on metabolic phenotype, formulation, dose, and duration, consistent with the heterogeneity observed in clinical studies.

### Safety Considerations for Supplemental CrIII

Gastrointestinal symptoms and headache are among the most frequently reported non-specific adverse events documented in randomized human trials using relatively common doses (approximately 200–1000 µg/day), although these events were reported in only a small number of the included studies [90, 106]. However, there are still some unresolved safety concerns that need to be handled carefully. These are: (i) signals of genotoxicity or carcinogenicity with some CrIII complexes in vitro and/or animals; (ii) limited knowledge on long-term, high dose safety data despite wide supplement use; and (iii) formulation-specific properties regarding bioavailability, intracellular accumulation, and potential presence of CrVI, notably for particulate or ‘nano’ sized preparations [10, 14, 102, 107].

Early biochemical studies demonstrated that CrIII binds to transferrin, the principal iron transport protein in plasma, forming complexes at its metal-binding sites and thereby potentially competing with Fe³⁺ in a concentration-dependent manner [108, 109]. Subsequent in vitro investigations confirmed that chromium reduces iron uptake by apotransferrin and may act as a more potent inhibitor of iron binding than aluminum under comparable conditions [110]. In vivo, chronic chromium administration at supraphysiological doses (1 mg/kg/day) in rats significantly decreased serum iron, total iron binding capacity, ferritin, and hemoglobin levels, consistent with impaired iron utilization [111]. Although these experimental doses exceed typical human supplementation ranges, clinical intakes up to 1000 µg/day have been reported, and the potential for chromium–iron interactions at the level of transferrin warrants consideration in the safety evaluation of high-dose supplementation. Lukaski et al. [112] reported a greater reduction in transferrin saturation in young men supplemented with chromium picolinate (24%) compared with chromium chloride or placebo (10–13%) during resistance training, although these changes occurred in the context of exercise-induced alterations in iron-related indices rather than a clearly isolated chromium effect. In contrast, Campbell et al. [113] observed no significant effects of high-dose chromium picolinate (~ 924 µg/day) on hematologic or iron status markers in older men. These findings indicate inconsistent outcomes across human studies, which may reflect differences in study populations, exercise protocols, baseline iron status, and chromium intake levels rather than a consistent dose-dependent relationship. Overall, the available evidence remains insufficient to determine whether CrIII supplementation meaningfully alters iron homeostasis; however, this issue warrants careful monitoring in high-dose or long-term human studies. From a regulatory-scientific standpoint, these factors provide grounds against the widespread nutritionally-based recommendation of a CrIII supplement. Rather, they make a case for a vigilant, phenotype-specific trial-level adoption of CrIII in obesity and diabetes research that would consider formulation/compound purity, regimen integrity, and long-term safety vigilance. The present evidence clearly distinguishes the severe toxicity and carcinogenicity of CrVI from the comparatively safer, yet not entirely risk-free, profile of CrIII. However, recent evaluations by major regulatory bodies and high-level reviews have raised substantial doubts regarding chromium essentiality, primarily due to the absence of clearly defined deficiency syndromes, the lack of reliable biomarkers of chromium status, and the modest and heterogeneous clinical outcomes observed in supplementation studies [16, 103]. Notably, although a potential molecular mechanism for CrIII action has been proposed through its interaction with mitochondrial ATP synthase [23], this mechanistic advance has yet to translate into consensus regarding clinical efficacy or regulatory endorsement of supplementation. In the setting of obesity and metabolic disease, CrIII should thus be viewed not as an essential nutrient but rather as a context-dependent pharmacological agent whose benefits will vary by metabolic phenotype, formulation, dose, and duration. This framework provides a consistent regulatory and scientific basis for understanding the potential and limits of chromium supplementation for human metabolic health.

## Conclusions

Chromium(III) should not be viewed solely as a direct enhancer of insulin signaling, but rather as a context-dependent modulator whose biological effects appear to emerge primarily under conditions of metabolic or physiological stress. Importantly, this interpretation is consistent with the pioneering observations of Mertz and Anderson [11], who demonstrated that chromium supplementation was most effective in experimental settings involving impaired glucose tolerance, nutritional insufficiency, or other metabolic perturbations. Contemporary findings in obesity, insulin resistance, oxidative stress, and heat-stress models broadly support this stress-responsive framework. Within this context, the proposed ATP synthase–AMPK/Nrf2/NF-κB network should be interpreted not as evidence of a universal chromium mechanism, but as a hypothesis-generating systems model potentially explaining why chromium responsiveness is more evident in metabolically challenged states than in healthy physiological conditions. Available experimental evidence permits, but does not yet validate, an integrated working model in which CrIII may influence mitochondrial energy sensing, redox regulation, and inflammatory tone under specific experimental conditions. Selected experimental studies suggest that certain CrIII formulations may modulate Nrf2/HO-1 signaling and attenuate NF-κB activity; however, these effects are highly dependent on formulation, dose, and experimental context and have not been consistently demonstrated in human studies. This review advances the field in three specific ways. First, it re-centers chromium biology on ATP synthase–AMPK signaling rather than classical insulin pathways, positioning CrIII within contemporary energy-sensing biology. Second, it integrates AMPK, Nrf2/HO-1, and NF-κB into a unified energy–redox–inflammation network, framing antioxidant, anti-inflammatory, and metabolic effects as mechanistically coupled rather than independent phenomena. Third, it provides a translational bridge by defining mechanism-linked biomarkers, including AMPK activation, Nrf2/HO-1 induction, and NF-κB suppression, as essential endpoints for future trials aimed at identifying responders and validating dose- and formulation-dependent target engagement. Moving forward, biomarker-anchored, phenotype-stratified clinical studies are required to clarify whether AMPK activation and Nrf2/HO-1 induction constitute reproducible mechanistic signatures of CrIII responsiveness in obesity and related metabolic disorders. Overall, current human evidence does not validate any specific molecular mechanism of CrIII action, and proposed pathways should be considered hypothesis-generating rather than mechanistically established. 

## Data Availability

No datasets were generated or analysed during the current study.

## References

[1] Barazzoni R, Gortan Cappellari G, Ragni M, Nisoli E (2018) Insulin resistance in obesity: an overview of fundamental alterations. Eat Weight Disord EWD 23:149–157. 10.1007/s40519-018-0481-6 29397563 10.1007/s40519-018-0481-6

[2] Ahmed B, Sultana R, Greene MW (2021) Adipose tissue and insulin resistance in obese. Biomed Pharmacother Biomedecine Pharmacother 137:111315. 10.1016/j.biopha.2021.111315 10.1016/j.biopha.2021.111315 33561645

[3] Cavaliere G, Cimmino F, Trinchese G et al (2023) From Obesity-Induced Low-Grade Inflammation to Lipotoxicity and Mitochondrial Dysfunction: Altered Multi-Crosstalk between Adipose Tissue and Metabolically Active Organs. Antioxidants 12:1172. 10.3390/antiox12061172 37371902 10.3390/antiox12061172 PMC10295335

[4] Salminen A, Hyttinen JMT, Kaarniranta K (2011) AMP-activated protein kinase inhibits NF-κB signaling and inflammation: impact on healthspan and lifespan. J Mol Med 89:667–676. 10.1007/s00109-011-0748-0 21431325 10.1007/s00109-011-0748-0 PMC3111671

[5] Petsouki E, Gerakopoulos V, Gianniou DD et al (2025) Involvement of NRF2 and AMPK signaling in aging and progeria: a digest. Redox Biol 85:103782. 10.1016/j.redox.2025.103782 40706292 10.1016/j.redox.2025.103782 PMC12309964

[6] Petsouki E, Cabrera SNS, Heiss EH (2022) AMPK and NRF2: Interactive players in the same team for cellular homeostasis? Free Radic Biol Med 190:75–93. 10.1016/j.freeradbiomed.2022.07.014 35918013 10.1016/j.freeradbiomed.2022.07.014

[7] Lau FC, Bagchi M, Sen CK, Bagchi D (2008) Nutrigenomic basis of beneficial effects of chromium(III) on obesity and diabetes. Mol Cell Biochem 317:1–10. 10.1007/s11010-008-9744-2 18636317 10.1007/s11010-008-9744-2

[8] Chen W-Y, Chen C-J, Liu C-H, Mao FC (2009) Chromium supplementation enhances insulin signalling in skeletal muscle of obese KK/HlJ diabetic mice. Diabetes Obes Metab 11:293–303. 10.1111/j.1463-1326.2008.00936.x 18721257 10.1111/j.1463-1326.2008.00936.x

[9] Lewicki S, Zdanowski R, Krzyżowska M et al (2014) The role of Chromium III in the organism and its possible use in diabetes and obesity treatment. Ann Agric Environ Med AAEM 21:331–335. 10.5604/1232-1966.1108599 24959784 10.5604/1232-1966.1108599

[10] Genchi G, Lauria G, Catalano A et al (2021) The Double Face of Metals: The Intriguing Case of Chromium. Appl Sci 11:638. 10.3390/app11020638

[11] Anderson RA, Polansky MM, Bryden NA, Canary JJ (1991) Supplemental-chromium effects on glucose, insulin, glucagon, and urinary chromium losses in subjects consuming controlled low-chromium diets. Am J Clin Nutr 54:909–916. 10.1093/ajcn/54.5.909 1951165 10.1093/ajcn/54.5.909

[12] Glinsmann WH, Mertz W (1966) Effect of trivalent chromium on glucose tolerance. Metabolism 15:510–520. 10.1016/0026-0495(66)90111-9 5933913 10.1016/0026-0495(66)90111-9

[13] Mertz W (1993) Chromium in human nutrition: a review. J Nutr 123:626–633. 10.1093/jn/123.4.626 8463863 10.1093/jn/123.4.626

[14] Vincent JB (2019) Effects of chromium supplementation on body composition, human and animal health, and insulin and glucose metabolism. Curr Opin Clin Nutr Metab Care 22:483. 10.1097/MCO.0000000000000604 31577642 10.1097/MCO.0000000000000604

[15] Henriksen C, Bügel S (2023) Chromium - a scoping review for Nordic Nutrition Recommendations 2023. Food Nutr Res 67. 10.29219/fnr.v67.10325 10.29219/fnr.v67.10325 PMC10710855 38084146

[16] Georgaki M-N, Tsokkou S, Keramas A et al (2024) Chromium supplementation and type 2 diabetes mellitus: an extensive systematic review. Environ Geochem Health 46:515. 10.1007/s10653-024-02297-5 39541030 10.1007/s10653-024-02297-5

[17] Asbaghi O, Fatemeh N, Mahnaz RK et al (2020) Effects of chromium supplementation on glycemic control in patients with type 2 diabetes: a systematic review and meta-analysis of randomized controlled trials. Pharmacol Res 161:105098. 10.1016/j.phrs.2020.105098 32730903 10.1016/j.phrs.2020.105098

[18] Tsang C, Taghizadeh M, Aghabagheri E et al (2019) A meta-analysis of the effect of chromium supplementation on anthropometric indices of subjects with overweight or obesity. Clin Obes 9:e12313. 10.1111/cob.12313 31115179 10.1111/cob.12313

[19] Yin RV, Phung OJ (2015) Effect of chromium supplementation on glycated hemoglobin and fasting plasma glucose in patients with diabetes mellitus. Nutr J 14:14. 10.1186/1475-2891-14-14 25971249 10.1186/1475-2891-14-14 PMC4430034

[20] Suksomboon N, Poolsup N, Yuwanakorn A (2014) Systematic review and meta-analysis of the efficacy and safety of chromium supplementation in diabetes. J Clin Pharm Ther 39:292–306. 10.1111/jcpt.12147 24635480 10.1111/jcpt.12147

[21] Costello RB, Dwyer JT, Bailey RL (2016) Chromium supplements for glycemic control in type 2 diabetes: limited evidence of effectiveness. Nutr Rev 74:455–468. 10.1093/nutrit/nuw011 27261273 10.1093/nutrit/nuw011 PMC5009459

[22] Asbaghi O, Naeini F, Ashtary-Larky D et al (2021) Effects of chromium supplementation on lipid profile in patients with type 2 diabetes: A systematic review and dose-response meta-analysis of randomized controlled trials. J Trace Elem Med Biol Organ Soc Min Trace Elem 66:126741. 10.1016/j.jtemb.2021.126741 10.1016/j.jtemb.2021.126741 33813266

[23] Wang H, Hu L, Li H et al (2023) Mitochondrial ATP synthase as a direct molecular target of chromium(III) to ameliorate hyperglycaemia stress. Nat Commun 14:1738. 10.1038/s41467-023-37351-w 36977671 10.1038/s41467-023-37351-w PMC10050403

[24] Gencoglu H, Orhan C, Sahin K (2024) Understanding Cr(III) Action on Mitochondrial ATP Synthase and AMPK Efficacy: Insights from Previous Studies-a Review. Biol Trace Elem Res 202:1325–1334. 10.1007/s12011-023-04010-6 38105318 10.1007/s12011-023-04010-6

[25] Tuzcu M, Sahin N, Orhan C et al (2011) Impact of chromium histidinate on high fat diet induced obesity in rats. Nutr Metab 8:28. 10.1186/1743-7075-8-28 10.1186/1743-7075-8-28 PMC3094204 21539728

[26] Sahin K, Tuzcu M, Orhan C et al (2012) The Effects of Chromium Picolinate and Chromium Histidinate Administration on NF-κB and Nrf2/HO-1 Pathway in the Brain of Diabetic Rats. Biol Trace Elem Res 150:291–296. 10.1007/s12011-012-9475-9 22790776 10.1007/s12011-012-9475-9

[27] Fotschki B, Ognik K, Fotschki J et al (2023) Chromium Nanoparticles Together with a Switch Away from High-Fat/Low-Fiber Dietary Habits Enhances the Pro-Healthy Regulation of Liver Lipid Metabolism and Inflammation in Obese Rats. Int J Mol Sci 24:2940. 10.3390/ijms24032940 36769261 10.3390/ijms24032940 PMC9918060

[28] de Mello AH, Costa AB, Engel JDG, Rezin GT (2018) Mitochondrial dysfunction in obesity. Life Sci 192:26–32. 10.1016/j.lfs.2017.11.019 29155300 10.1016/j.lfs.2017.11.019

[29] Mattos Pereira V, Nair S (2024) Targeting Mitochondrial ATP-Synthase: Evolving Role of Chromium as a Regulator of Carbohydrate and Fat Metabolism. Biol Trace Elem Res 202:1318–1324. 10.1007/s12011-023-04017-z 38133723 10.1007/s12011-023-04017-z

[30] Day EA, Ford RJ, Steinberg GR (2017) AMPK as a Therapeutic Target for Treating Metabolic Diseases. Trends Endocrinol Metab TEM 28:545–560. 10.1016/j.tem.2017.05.004 28647324 10.1016/j.tem.2017.05.004

[31] Oriquat G, Masoud IM, Kamel MA et al (2023) The Anti-Obesity and Anti-Steatotic Effects of Chrysin in a Rat Model of Obesity Mediated through Modulating the Hepatic AMPK/mTOR/lipogenesis Pathways. Molecules 28:1734. 10.3390/molecules28041734 36838721 10.3390/molecules28041734 PMC9962978

[32] Wu S, Zou M-H (2020) AMPK, Mitochondrial Function, and Cardiovascular Disease. Int J Mol Sci 21:4987. 10.3390/ijms21144987 32679729 10.3390/ijms21144987 PMC7404275

[33] Herzig S, Shaw RJ (2018) AMPK: guardian of metabolism and mitochondrial homeostasis. Nat Rev Mol Cell Biol 19:121–135. 10.1038/nrm.2017.95 28974774 10.1038/nrm.2017.95 PMC5780224

[34] Garcia D, Shaw RJ (2017) AMPK: Mechanisms of Cellular Energy Sensing and Restoration of Metabolic Balance. Mol Cell 66:789–800. 10.1016/j.molcel.2017.05.032 28622524 10.1016/j.molcel.2017.05.032 PMC5553560

[35] Jeon S-M (2016) Regulation and function of AMPK in physiology and diseases. Exp Mol Med 48:e245–e245. 10.1038/emm.2016.81 27416781 10.1038/emm.2016.81 PMC4973318

[36] Park YM, Lee HY, Shin DY et al (2025) Chestnut (*Castanea crenata* S. et Z.) inner shell-A by-product promotes anti-obesity by regulating adipogenesis and lipogenesis-related genes and the AMPK/ACC signaling pathway in high-fat diet-induced obese mice. Heliyon 11:e42586. 10.1016/j.heliyon.2025.e42586 40066053 10.1016/j.heliyon.2025.e42586 PMC11891676

[37] Steinberg GR, Hardie DG (2023) New insights into activation and function of the AMPK. Nat Rev Mol Cell Biol 24:255–272. 10.1038/s41580-022-00547-x 36316383 10.1038/s41580-022-00547-x

[38] Wu L, Zhang L, Li B et al (2018) AMP-Activated Protein Kinase (AMPK) Regulates Energy Metabolism through Modulating Thermogenesis in Adipose Tissue. Front Physiol 9:122. 10.3389/fphys.2018.00122 29515462 10.3389/fphys.2018.00122 PMC5826329

[39] Hoffman NJ, Penque BA, Habegger KM et al (2014) Chromium enhances insulin responsiveness via AMPK. J Nutr Biochem 25:565–572. 10.1016/j.jnutbio.2014.01.007 24725432 10.1016/j.jnutbio.2014.01.007 PMC4030743

[40] Hao J, Hao C, Zhang L et al (2015) OM2, a Novel Oligomannuronate-Chromium(III) Complex, Promotes Mitochondrial Biogenesis and Lipid Metabolism in 3T3-L1 Adipocytes via the AMPK-PGC1α Pathway. PLoS ONE 10:e0131930. 10.1371/journal.pone.0131930 26176781 10.1371/journal.pone.0131930 PMC4503612

[41] Hao C, Hao J, Wang W et al (2011) Insulin sensitizing effects of oligomannuronate-chromium (III) complexes in C2C12 skeletal muscle cells. PLoS ONE 6:e24598. 10.1371/journal.pone.0024598 21935427 10.1371/journal.pone.0024598 PMC3174176

[42] Ngo V, Duennwald ML (2022) Nrf2 and Oxidative Stress: A General Overview of Mechanisms and Implications in Human Disease. Antioxidants 11:2345. 10.3390/antiox11122345 36552553 10.3390/antiox11122345 PMC9774434

[43] Tkaczenko H, Kurhaluk N (2025) Antioxidant-Rich Functional Foods and Exercise: Unlocking Metabolic Health Through Nrf2 and Related Pathways. Int J Mol Sci 26:1098. 10.3390/ijms26031098 39940866 10.3390/ijms26031098 PMC11817741

[44] Tebay LE, Robertson H, Durant ST et al (2015) Mechanisms of activation of the transcription factor Nrf2 by redox stressors, nutrient cues, and energy status and the pathways through which it attenuates degenerative disease. Free Radic Biol Med 88:108–146. 10.1016/j.freeradbiomed.2015.06.021 26122708 10.1016/j.freeradbiomed.2015.06.021 PMC4659505

[45] Saha S, Buttari B, Panieri E et al (2020) An Overview of Nrf2 Signaling Pathway and Its Role in Inflammation. Molecules 25:5474. 10.3390/molecules25225474 33238435 10.3390/molecules25225474 PMC7700122

[46] Alonso-Piñeiro JA, Gonzalez-Rovira A, Sánchez-Gomar I et al (2021) Nrf2 and Heme Oxygenase-1 Involvement in Atherosclerosis Related Oxidative Stress. Antioxidants 10:1463. 10.3390/antiox10091463 34573095 10.3390/antiox10091463 PMC8466960

[47] Xu Y, Bai L, Yang X et al (2024) Recent advances in anti-inflammation via AMPK activation. Heliyon 10:e33670. 10.1016/j.heliyon.2024.e33670 39040381 10.1016/j.heliyon.2024.e33670 PMC11261115

[48] Yang Z, Kahn BB, Shi H, Xue B (2010) Macrophage α1 AMP-activated Protein Kinase (α1AMPK) Antagonizes Fatty Acid-induced Inflammation through SIRT1*. J Biol Chem 285:19051–19059. 10.1074/jbc.M110.123620 20421294 10.1074/jbc.M110.123620 PMC2885183

[49] Selcuk MY, Aygen B, Dogukan A et al (2012) Chromium picolinate and chromium histidinate protects against renal dysfunction by modulation of NF-κB pathway in high-fat diet fed and Streptozotocin-induced diabetic rats. Nutr Metab 9:30. 10.1186/1743-7075-9-30 10.1186/1743-7075-9-30 PMC3348071 22483164

[50] Anitha KN, Mutahir M, Dhadde SB et al (2025) Neuroprotective Potential of Chromium-D-Phenylalanine: Insights from Molecular Docking, Dynamics and Rotenone-Induced Parkinsonism in Rats. Biol Trace Elem Res. 10.1007/s12011-025-04671-5 40439854 10.1007/s12011-025-04671-5

[51] Gholami A, Sohrabi M, Baradaran HR, Hariri M (2025) Effect of Chromium Supplementation on Serum Levels of Inflammatory Mediators: An Updated Systematic Review and Meta-analysis on Randomized Clinical Trials. Biol Trace Elem Res 203:4065–4078. 10.1007/s12011-024-04486-w 39671146 10.1007/s12011-024-04486-w

[52] Joo MS, Kim WD, Lee KY et al (2016) AMPK Facilitates Nuclear Accumulation of Nrf2 by Phosphorylating at Serine 550. Mol Cell Biol 36:1931–1942. 10.1128/MCB.00118-16 27161318 10.1128/MCB.00118-16 PMC4936058

[53] Fischhuber K, Matzinger M, Heiss EH (2020) AMPK Enhances Transcription of Selected Nrf2 Target Genes via Negative Regulation of Bach1. Front Cell Dev Biol 8. 10.3389/fcell.2020.00628 10.3389/fcell.2020.00628 PMC7372114 32760724

[54] Zimmermann K, Baldinger J, Mayerhofer B et al (2015) Activated AMPK boosts the Nrf2/HO-1 signaling axis–A role for the unfolded protein response. Free Radic Biol Med 88:417–426. 10.1016/j.freeradbiomed.2015.03.030 25843659 10.1016/j.freeradbiomed.2015.03.030 PMC4568300

[55] Yu CHJ, Rupasinghe HPV (2025) Elucidating the mechanistic interplay of AMPK and Nrf2 in MASLD: A focus on dietary phytochemical modulation of lipid and redox homeostasis. Biochem Biophys Res Commun 777:152300. 10.1016/j.bbrc.2025.152300 40633499 10.1016/j.bbrc.2025.152300

[56] Mo C, Wang L, Zhang J et al (2014) The crosstalk between Nrf2 and AMPK signal pathways is important for the anti-inflammatory effect of berberine in LPS-stimulated macrophages and endotoxin-shocked mice. Antioxid Redox Signal 20:574–588. 10.1089/ars.2012.5116 23875776 10.1089/ars.2012.5116 PMC3901384

[57] Catrysse L, van Loo G (2017) Inflammation and the Metabolic Syndrome: The Tissue-Specific Functions of NF-κB. Trends Cell Biol 27:417–429. 10.1016/j.tcb.2017.01.006 28237661 10.1016/j.tcb.2017.01.006

[58] Suren Garg S, Kushwaha K, Dubey R, Gupta J (2023) Association between obesity, inflammation and insulin resistance: Insights into signaling pathways and therapeutic interventions. Diabetes Res Clin Pract 200:110691. 10.1016/j.diabres.2023.110691 37150407 10.1016/j.diabres.2023.110691

[59] Gkrinia EMM, Belančić A (2025) The Mechanisms of Chronic Inflammation in Obesity and Potential Therapeutic Strategies: A Narrative Review. Curr Issues Mol Biol 47. 10.3390/cimb47050357 10.3390/cimb47050357 PMC12110701 40699756

[60] Xu S, Xi J, Wu T, Wang Z (2023) The Role of Adipocyte Endoplasmic Reticulum Stress in Obese Adipose Tissue Dysfunction: A Review. Int J Gen Med 16:4405–4418. 10.2147/IJGM.S428482 37789878 10.2147/IJGM.S428482 PMC10543758

[61] Akhter N, Wilson A, Arefanian H et al (2023) Endoplasmic Reticulum Stress Promotes the Expression of TNF-α in THP-1 Cells by Mechanisms Involving ROS/CHOP/HIF-1α and MAPK/NF-κB Pathways. Int J Mol Sci 24:15186. 10.3390/ijms242015186 37894865 10.3390/ijms242015186 PMC10606873

[62] Griffin MJ (2022) On the Immunometabolic Role of NF-κB in Adipocytes. Immunometabolism 4:e220003. 10.20900/immunometab20220003 35251704 10.20900/immunometab20220003 PMC8893669

[63] Kim S, Joe Y, Jeong SO et al (2014) Endoplasmic reticulum stress is sufficient for the induction of IL-1β production via activation of the NF-κB and inflammasome pathways. Innate Immun 20:799–815. 10.1177/1753425913508593 24217221 10.1177/1753425913508593

[64] Park JE, Kang E, Han JS (2022) HM-chromanone attenuates TNF-α-mediated inflammation and insulin resistance by controlling JNK activation and NF-κB pathway in 3T3-L1 adipocytes. Eur J Pharmacol 921:174884. 10.1016/j.ejphar.2022.174884 35288193 10.1016/j.ejphar.2022.174884

[65] Baker RG, Hayden MS, Ghosh S (2011) NF-κB, inflammation, and metabolic disease. Cell Metab 13:11–22. 10.1016/j.cmet.2010.12.008 21195345 10.1016/j.cmet.2010.12.008 PMC3040418

[66] Mobeen A, Joshi S, Fatima F et al (2025) NF-κB signaling is the major inflammatory pathway for inducing insulin resistance. 3 Biotech 15:47. 10.1007/s13205-024-04202-4 39845928 10.1007/s13205-024-04202-4 PMC11747027

[67] Ren Y, Zhao H, Yin C et al (2022) Adipokines, Hepatokines and Myokines: Focus on Their Role and Molecular Mechanisms in Adipose Tissue Inflammation. Front Endocrinol 13:873699. 10.3389/fendo.2022.873699 10.3389/fendo.2022.873699 PMC9329830 35909571

[68] Cai D, Yuan M, Frantz DF et al (2005) Local and systemic insulin resistance resulting from hepatic activation of IKK-beta and NF-kappaB. Nat Med 11:183–190. 10.1038/nm1166 15685173 10.1038/nm1166 PMC1440292

[69] Crasan I-M, Tanase M, Delia CE et al (2025) Metaflammation’s Role in Systemic Dysfunction in Obesity: A Comprehensive Review. Int J Mol Sci 26:10445. 10.3390/ijms262110445 41226484 10.3390/ijms262110445 PMC12607572

[70] Li M, Hou Y, Chen Y et al (2024) Palmitic acid promotes miRNA release from adipocyte exosomes by activating NF-κB/ER stress. Nutr Diabetes 14:75. 10.1038/s41387-024-00334-x 39271650 10.1038/s41387-024-00334-x PMC11399118

[71] Lee D-K, Kim T, Byeon J et al (2022) REDD1 promotes obesity-induced metabolic dysfunction via atypical NF-κB activation. Nat Commun 13:6303. 10.1038/s41467-022-34110-1 36272977 10.1038/s41467-022-34110-1 PMC9588012

[72] Zhao Y, Zhou M, Shang Y et al (2024) Effects of co-supplementation of chromium and magnesium on metabolic profiles, inflammation, and oxidative stress in impaired glucose tolerance. Diab Vasc Dis Res 21:14791641241228156. 10.1177/14791641241228156 38228168 10.1177/14791641241228156 PMC10798099

[73] Morvaridzadeh M, Estêvão MD, Qorbani M et al (2022) The effect of chromium intake on oxidative stress parameters: A systematic review and meta-analysis. J Trace Elem Med Biol 69:126879. 10.1016/j.jtemb.2021.126879 34710707 10.1016/j.jtemb.2021.126879

[74] Li Q, Yuan Y, Qiao M et al (2026) Antioxidative stress effect of chromium-chelated pea (Pisum sativum L.) peptides through the Keap1/Nrf2 pathway. J Sci Food Agric 106:1674–1684. 10.1002/jsfa.70293 41312787 10.1002/jsfa.70293

[75] Lewandowska H, Chen Z, Marszałek K et al (2025) Effective Antioxidants as Plausible Ligands in Chromium(III) Supplementation: How Complexation Modulates Catechol-Based Polyphenols. Molecules 30:4467. 10.3390/molecules30224467 41302522 10.3390/molecules30224467 PMC12655245

[76] Peng M, Yang X (2015) Controlling diabetes by chromium complexes: The role of the ligands. J Inorg Biochem 146:97–103. 10.1016/j.jinorgbio.2015.01.002 25631328 10.1016/j.jinorgbio.2015.01.002

[77] Li P, Li M, Lou X et al (2022) Evaluation of Hypoglycemic Activity and Sub-Acute Toxicity of the Novel Biochanin A–Chromium(III) Complex. Molecules 27:5786. 10.3390/molecules27185786 36144522 10.3390/molecules27185786 PMC9504010

[78] Zhao P, Wang J, Ma H et al (2009) A newly synthetic chromium complex-chromium (D-phenylalanine)3 activates AMP-activated protein kinase and stimulates glucose transport. Biochem Pharmacol 77:1002–1010. 10.1016/j.bcp.2008.11.018 19073152 10.1016/j.bcp.2008.11.018

[79] Dong F, Kandadi MR, Ren J, Sreejayan N (2008) Chromium (D-phenylalanine)3 supplementation alters glucose disposal, insulin signaling, and glucose transporter-4 membrane translocation in insulin-resistant mice. J Nutr 138:1846–1851. 10.1093/jn/138.10.1846 18806091 10.1093/jn/138.10.1846

[80] Kandadi MR, Unnikrishnan MK, Warrier AKS et al (2011) Chromium (D-phenylalanine)3 alleviates high fat-induced insulin resistance and lipid abnormalities. J Inorg Biochem 105:58–62. 10.1016/j.jinorgbio.2010.09.008 21134603 10.1016/j.jinorgbio.2010.09.008 PMC3046066

[81] Sahin K, Kucuk O, Orhan C et al (2021) Effects of supplementing different chromium histidinate complexes on glucose and lipid metabolism and related protein expressions in rats fed a high-fat diet. J Trace Elem Med Biol Organ Soc Min Trace Elem 65:126723. 10.1016/j.jtemb.2021.126723 10.1016/j.jtemb.2021.126723 33508549

[82] Sreejayan N, Dong F, Kandadi MR et al (2008) Chromium alleviates glucose intolerance, insulin resistance, and hepatic ER stress in obese mice. Obesity 16:1331–1337. 10.1038/oby.2008.217 18388893 10.1038/oby.2008.217

[83] Wang ZQ, Zhang XH, Russell JC et al (2006) Chromium picolinate enhances skeletal muscle cellular insulin signaling in vivo in obese, insulin-resistant JCR:LA-cp rats. J Nutr 136:415–420. 10.1093/jn/136.2.415 16424121 10.1093/jn/136.2.415

[84] Feng W, Liu Y, Fei F et al (2018) Improvement of high-glucose and insulin resistance of chromium malate in 3T3-L1 adipocytes by glucose uptake and insulin sensitivity signaling pathways and its mechanism. RSC Adv 9:114–127. 10.1039/C8RA07470D 35521592 10.1039/c8ra07470d PMC9059288

[85] Wang H, Kruszewski A, Brautigan DL (2005) Cellular chromium enhances activation of insulin receptor kinase. Biochemistry 44:8167–8175. 10.1021/bi0473152 15924436 10.1021/bi0473152

[86] Mackowiak P, Krejpcio Z, Sassek M et al (2010) Evaluation of insulin binding and signaling activity of newly synthesized chromium(III) complexes in vitro. Mol Med Rep 3:347–353. 10.3892/mmr_00000264 21472246 10.3892/mmr_00000264

[87] Akhtar A, Dhaliwal J, Saroj P et al (2020) Chromium picolinate attenuates cognitive deficit in ICV-STZ rat paradigm of sporadic Alzheimer’s-like dementia via targeting neuroinflammatory and IRS-1/PI3K/AKT/GSK-3β pathway. Inflammopharmacology 28:385–400. 10.1007/s10787-019-00681-7 31898080 10.1007/s10787-019-00681-7

[88] Kooshki F, Tutunchi H, Vajdi M et al (2021) A Comprehensive insight into the effect of chromium supplementation on oxidative stress indices in diabetes mellitus: A systematic review. Clin Exp Pharmacol Physiol 48:291–309. 10.1111/1440-1681.13462 33462845 10.1111/1440-1681.13462

[89] Monfared V, Rashin H, Malekinejad S et al (2025) The effect of chromium supplementation on cardio-metabolic risk factors in overweight and obese patients. A systematic review and meta-analysis of randomized controlled trial. J Trace Elem Med Biol Organ Soc Min Trace Elem GMS 89:127645. 10.1016/j.jtemb.2025.127645 10.1016/j.jtemb.2025.127645 40245649

[90] Onakpoya I, Posadzki P, Ernst E (2013) Chromium supplementation in overweight and obesity: a systematic review and meta-analysis of randomized clinical trials. Obes Rev Off J Int Assoc Study Obes 14:496–507. 10.1111/obr.12026 10.1111/obr.12026 23495911

[91] Huang H, Chen G, Dong Y et al (2018) Chromium supplementation for adjuvant treatment of type 2 diabetes mellitus: Results from a pooled analysis. Mol Nutr Food Res 62. 10.1002/mnfr.201700438 10.1002/mnfr.201700438 28677892

[92] Zhang X, Cui L, Chen B et al (2021) Effect of chromium supplementation on hs-CRP, TNF-α and IL-6 as risk factor for cardiovascular diseases: A meta-analysis of randomized-controlled trials. Complement Ther Clin Pract 42:101291. 10.1016/j.ctcp.2020.101291 33321447 10.1016/j.ctcp.2020.101291

[93] Liu F, Cottrell JJ, Wijesiriwardana U et al (2017) Effects of chromium supplementation on physiology, feed intake, and insulin related metabolism in growing pigs subjected to heat stress. Transl Anim Sci 1:116–125. 10.2527/tas2017.0014 32704634 10.2527/tas2017.0014 PMC7205331

[94] Molz P, Molz WA, Dallemole DR et al (2021) Potential Ameliorative Effects of Chromium Supplementation on Glucose Metabolism, Obesity, and Genomic Stability in Prediabetic Rat Model. Biol Trace Elem Res 199:1893–1899. 10.1007/s12011-020-02299-1 32710349 10.1007/s12011-020-02299-1

[95] Piray A, Foroutanifar S (2022) Chromium Supplementation on the Growth Performance, Carcass Traits, Blood Constituents, and Immune Competence of Broiler Chickens Under Heat Stress: a Systematic Review and Dose–Response Meta-analysis. Biol Trace Elem Res 200:2876–2888. 10.1007/s12011-021-02885-x 34417722 10.1007/s12011-021-02885-x

[96] Vincent JB (2024) Is chromium(III) supplementation beneficial for dietary rodent models of prediabetes? J Trace Elem Med Biol 85:127482. 10.1016/j.jtemb.2024.127482 38861777 10.1016/j.jtemb.2024.127482

[97] Vincent JB (2014) Is chromium pharmacologically relevant? J Trace Elem Med Biol 28:397–405. 10.1016/j.jtemb.2014.06.020 25043953 10.1016/j.jtemb.2014.06.020

[98] Talab AT, Abdollahzad H, Nachvak SM et al (2020) Effects of Chromium Picolinate Supplementation on Cardiometabolic Biomarkers in Patients with Type 2 Diabetes Mellitus: a Randomized Clinical Trial. Clin Nutr Res 9:97–106. 10.7762/cnr.2020.9.2.97 32395440 10.7762/cnr.2020.9.2.97 PMC7192664

[99] Moradi F, Kooshki F, Nokhostin F et al (2021) A pilot study of the effects of chromium picolinate supplementation on serum fetuin-A, metabolic and inflammatory factors in patients with nonalcoholic fatty liver disease: A double-blind, placebo-controlled trial. J Trace Elem Med Biol Organ Soc Min Trace Elem 63:126659. 10.1016/j.jtemb.2020.126659 10.1016/j.jtemb.2020.126659 33045675

[100] Hossini H, Shafie B, Niri AD et al (2022) A comprehensive review on human health effects of chromium: insights on induced toxicity. Environ Sci Pollut Res Int 29:70686–70705. 10.1007/s11356-022-22705-6 36042133 10.1007/s11356-022-22705-6

[101] Sawicka E, Jurkowska K, Piwowar A (2021) Chromium (III) and chromium (VI) as important players in the induction of genotoxicity - current view. Ann Agric Environ Med AAEM 28:1–10. 10.26444/aaem/118228 33775062 10.26444/aaem/118228

[102] Schumacher P, Fischer F, Sann J et al (2022) Impact of Nano- and Micro-Sized Chromium(III) Particles on Cytotoxicity and Gene Expression Profiles Related to Genomic Stability in Human Keratinocytes and Alveolar Epithelial Cells. Nanomaterials 12:1294. 10.3390/nano12081294 35458002 10.3390/nano12081294 PMC9029936

[103] EFSA Panel on Dietetic Products, Nutrition and Allergies (NDA) (2014) Scientific Opinion on Dietary Reference Values for chromium. EFSA J 12:3845. 10.2903/j.efsa.2014.3845 42087961 10.2903/j.efsa.2014.3845 PMC13136993

[104] Institute of Medicine (US) Panel on Micronutrients (2001) Dietary Reference Intakes for. In: Vitamin A, Vitamin K (eds) Arsenic, Boron, Chromium, Copper, Iodine, Iron, Manganese, Molybdenum, Nickel, Silicon, Vanadium, and Zinc. National Academies Press (US), Washington (DC) 25057538

[105] Maret W (2019) Chromium Supplementation in Human Health, Metabolic Syndrome, and Diabetes. Met Ions Life Sci 19./books/9783110527872/9783110527872-015/9783110527872-015.xml10.1515/9783110527872-015 10.1515/9783110527872-015 30855110

[106] Tian H, Guo X, Wang X et al (2013) Chromium picolinate supplementation for overweight or obese adults. Cochrane Database Syst Rev 2013:CD010063. 10.1002/14651858.CD010063.pub2 10.1002/14651858.CD010063.pub2 PMC7433292 24293292

[107] DesMarais TL, Costa M (2019) Mechanisms of Chromium-Induced Toxicity. Curr Opin Toxicol 14:1–7. 10.1016/j.cotox.2019.05.003 31511838 10.1016/j.cotox.2019.05.003 PMC6737927

[108] Hopkins LL, Schwarz K (1964) Chromium (III) binding to serum proteins, specifically siderophilin. Biochim Biophys Acta BBA - Gen Subj 90:484–491. 10.1016/0304-4165(64)90228-4 10.1016/0304-4165(64)90228-4 14237857

[109] Aisen P, Aasa R, Redfield AG (1969) The Chromium, Manganese, and Cobalt Complexes of Transferrin. J Biol Chem 244:4628–4633. 10.1016/S0021-9258(18)93670-7 4309148

[110] Moshtaghie AA, Ani M, Bazrafshan MR (1992) Comaprative binding study of aluminum and chromium to human transferrin. Biol Trace Elem Res 32:39–46. 10.1007/BF02784585 1375080 10.1007/BF02784585

[111] Ani M, Moshtaghie AA (1992) The effect of chromium on parameters related to iron metabolism. Biol Trace Elem Res 32:57–64. 10.1007/BF02784588 1375087 10.1007/BF02784588

[112] Lukaski H, Bolonchuk W, Siders W, Milne D (1996) Chromium supplementation and resistance training: effects on body composition, strength, and trace element status of men. Am J Clin Nutr 63:954–965. 10.1093/ajcn/63.6.954 8644693 10.1093/ajcn/63.6.954

[113] Campbell WW, Beard JL, Joseph LJ et al (1997) Chromium picolinate supplementation and resistive training by older men: effects on iron-status and hematologic indexes. Am J Clin Nutr 66:944–949. 10.1093/ajcn/66.4.944 9322572 10.1093/ajcn/66.4.944

[114] Dworzański W, Cholewińska E, Fotschki B et al (2022) Oxidative, epigenetic changes and fermentation processes in the intestine of rats fed high-fat diets supplemented with various chromium forms. Sci Rep 12:9817. 10.1038/s41598-022-13328-5 35701510 10.1038/s41598-022-13328-5 PMC9198011

